# Umbelliprenin induces autophagy and apoptosis while inhibits cancer cell stemness in pancreatic cancer cells

**DOI:** 10.1002/cam4.6170

**Published:** 2023-07-06

**Authors:** Hongcheng Wang, Yongzhi Liu, Yiwei Wang, Ting Xu, Guanggai Xia, Xinyu Huang

**Affiliations:** ^1^ Department of Hepatobiliary and Pancreatic Surgery Sixth People's Hospital Affiliated Shanghai Jiao Tong University Shanghai China; ^2^ Department of General Surgery The Second People’ Hospital of Kashgar, 1S Kashgar Xinjiang China; ^3^ Affiliated Xiaoshan Hospital Hangzhou Normal University Zhejiang China

**Keywords:** Akt/mTOR signaling pathway, apoptosis, autophagy, cancer stem cells, pancreatic cancer

## Abstract

**Background:**

Umbelliprenin is a sesquiterpene coumarin isolated from *Artemisia absinthium* L. and shows antitumor effects in various cancers by inducing apoptosis. However, the antitumor effect of umbelliprenin in human pancreatic cancer has not been clarified.

**Methods:**

The antitumor effects were determined by MTT and AnnexinV/PI double staining assay in vitro and xenograft mice in vivo. Autophagy was determined via immunofluorescence analysis. Apoptotic or autophagic related proteins were measured by immunoblotting. The pancreatic cancer cell stemness were determined by mammosphere formation and ALDEFLUOR assay.

**Results:**

It revealed that umbelliprenin inhibited pancreatic cancer cell proliferation in vitro and pancreatic cancer tumor growth in vivo. Moreover, umbelliprenin induced pancreatic cancer cell BxPC3 apoptosis and autophagy as evidenced by upregulated apoptosis and autophagy‐ related protein expression (*p* < 0.01). Blocking autophagy by 3‐MA or Atg7 knockout enhanced umbelliprenin‐induced apoptosis (*p* < 0.05). Umbelliprenin also reduced pancreatic cancer cell stemness by reducing Oct4, Nanog, and Sox2 mRNA levels (*p* < 0.01). Mechanistically, umbelliprenin greatly inhibited Akt/mTOR and Notch1 signal pathway.

**Conclusion:**

Umbelliprenin may be a novel therapeutic approach for pancreatic cancer treatment.

## 1 INTRODUCTION

Pancreatic cancer is one of the most highly lethal malignancy cancers in the world with a 5‐year overall survival rate below 5%.^1^ The mainstay of chemotherapy combinations such as FOLFIRNOX and gemcitabine/nab‐paclitaxel only slightly improve pancreatic cancer patients' survival rates but have obvious side effects.^2^, ^3^ However, other treatment modalities are extremely lacking. Therefore, the development of novel anti‐pancreatic cancer agents with fewer side effects are urgently needed.

Pancreatic cancer contains various types of cells including a distinct subpopulation of pancreatic cancer stem cells (CSCs). Pancreatic CSCs contribute to tumor cell self‐renewal, differentiation, cancer recurrence, metastasis, and therapy resistance.^4^, ^5^ Markers that used to identify pancreatic CSCs include SOX2, OCT4, NANOG, ALDH1, CD24, CD44, and ESA.^6^, ^7^ Pancreatic CSCs are regulated by aberrant activation of signal pathways like NOTCH, WNT, and β‐catenin.^8^, ^9^ Therefore, targeting these signal pathways may provide new therapeutic strategies in the treatment of pancreatic cancer.

In recent years, medicinal plants have grabbed great attention for treating diseases such as cancer.^10^, ^11^ *Artemisia absinthium* L. (Chinese name: Zhong Ya Ku Hao), is widely distributed in Asia, Europe, and North America and used as folk medicine to treat anorexia, parasitic diseases, bacterial infection, and cancer.^12^, ^13^, ^14^ Additionally, *A. absinthium* extracts have been reported to inhibit breast cancer cell proliferation.^15^, ^16^ The methanolic extract of *A. absinthium* was reported to induce DNA damage and apoptosis in human colon cancer cells.^17^ Umbelliprenin is a 7‐prenyloxycoumarin extracted from *A. absinthium*, which shows antitumor effects in various cancer cell lines. Umbelliprenin inhibited human melanoma cell proliferation by inducing apoptosis and cell‐cycle arrest.^18^ In addition, umbelliprenin can inhibit cell migration and invasion in prostate cancer, gastric cancer, colorectal cancer, ovary cancer, and lung cancer.^15^, ^16^, ^18^, ^19^, ^20^, ^21^ However, the anticancer effect of umbelliprenin in pancreatic cancer have not been clarified yet.

Autophagy is a tightly orchestrated self‐digestion process that sequesters misfolded proteins and damaged organelles into autophagosomes and fuses with lysosomes for degradation.^22^ Serval upstream regulators like Akt/ mTOR, AMPK, and ROS can directly mediate autophagosome formation.^23^ Upregulated autophagy has been reported to promote tumor growth and help tumor cell survival from various stresses like starvation, radiotherapy, chemotherapy, or other anticancer agents. However, whether umbelliprenin could trigger autophagy in pancreatic cancer and the underlying mechanisms are still not clearly determined.

In the current study, we examined the anticancer role of umbelliprenin in pancreatic cancer and investigated its underlying mechanisms involving Akt/mTOR and Notch1 signaling pathways to regulate apoptosis, autophagy, and cancer cell stemness.

## 2 MATERIALS AND METHODS

### 2.1 Chemicals and antibodies

**FIGURE 1:**
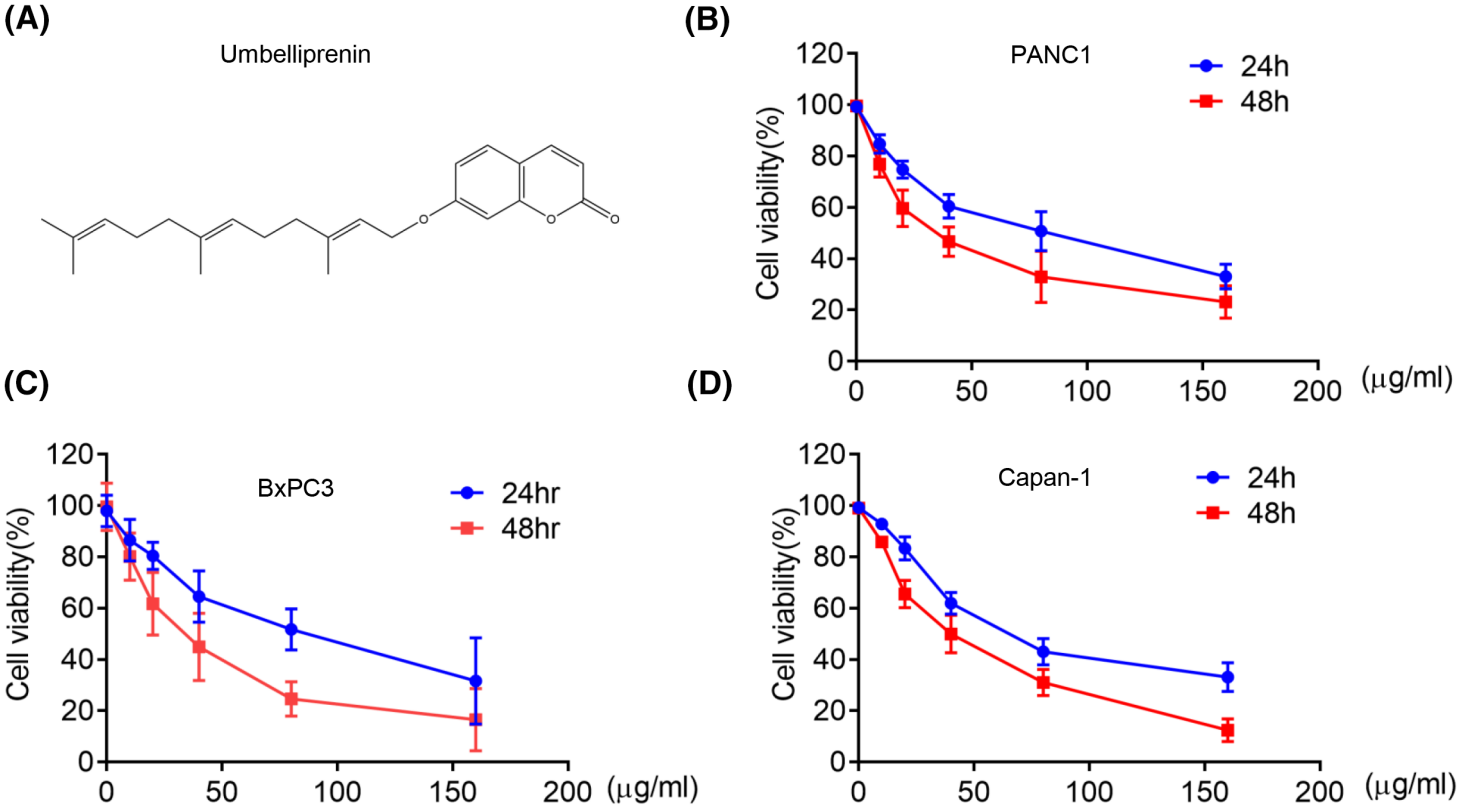
Umbelliprenin inhibited pancreatic cancer cell viability. (A) The chemical structure umbelliprenin. (B) PANC1, (C) BxPC3, and (D) Capan‐1 cells were treated with umbelliprenin (0–160 μg/mL) for 24 or 48 h and measured by using MTT assay (*n* = 3).

Umbelliprenin (Figure 1A) was obtained from Sigma‐Aldrich. 3‐methyladenine (3‐MA) and MTT were purchased from Sigma; Annex‐V Apoptosis Detection Kit was purchased from BD Biosciences; lipofectamine 3000 was from Invitrogen.The antibodies were used as follows: β‐actin, β‐catenin, caspase‐3, and ‐8, cleaved caspase ‐3and ‐8, Bax, Bcl‐2, LC3, p62, mTOR, p‐mTOR(Ser2448), AKT, p‐AKT(Ser473), SUFU, Shh, and Notch1‐4 were from Cell Signaling Technology; Vps34, Beclin1, Atg7 were from Abcam.

### 2.2 Cell culture and MTT assay

PANC‐1, Capan‐1, and BxPC3 (ATCC) pancreatic cancer cell lines were maintained in DMEM medium with 10% FBS and 1% penicillin/streptomycin in a humidified incubator containing 5% CO2 at 37°C.

For MTT assay, 1 × 10^4^ exponentially growing Capan‐1, BxPC3, and PANC‐1 cells were seeded into 96‐well culture plates overnight. The medium was then exchanged with DMEM containing different concentrations of umbelliprenin (10, 20, 40,80, and 160 μg/mL) and DMSO groups as a control for 24 or 48 h. After that, MTT assay was conducted by using MTT in accordance with the manufacturer's instructions and measured at 540 nm with a microplate reader (Thermo Fisher Scientific, Inc.).

### 2.3 Apoptosis Assay

To investigate the extent of umbelliprenin‐induced apoptosis in BXPC‐3 cells, Annexin V/PI staining assay was performed. Briefly, BXPC‐3 cells (1 × 10^5^ cells/well) were treated with different concentrations of umbelliprenin (0, 10, 20, and 40 μg/mL) for 24 h. Then the cells were harvested, washed, and stained with Annexin V‐FITC/PI Apoptosis Detection kit (BD Biosciences). After 20 min incubation, the flow cytometry analysis was performed (Beckman Coulter, Inc.).

### 2.4 Mammosphere assay

BxPC3 cells (500 cells/cm^2^) were seeded into 6‐well plates in Mammosphere Media containing B27 supplement and EGF (20 ng/mL; PeproTech) in the presence or absence of 10 μg/mL of umbelliprenin for 5 days.

### 2.5 Aldefluor assay

ALDEFLOUR™ assay kits (Stemcell Technologies, Inc.) were used for aldefluor assay. BxPC3 cells were seeded in 6‐well plates and treated with/without 10 μg/mL of umbelliprenin for 5 days. Then 1 × 10^6^ cell were resuspended in ALDEFLUOR buffer containing 5 μL BAAA and/or 5 μL DEAB for 40 min at 37°C. FACS analysis were conducted by flow cytometer (BD Biosciences) and analyzed by FlowJo software (Version 10; FlowJo LLC).

### 2.6 RT‐PCR

BxPC3 cells were seeded in 6‐well plates (1 × 10^6^/well) and treated with/without 10 μg/mL of umbelliprenin for 5 days. Then total RNA was extracted by TRIzol® reagent and quantified by NanoDrop spectrophotometer. Reverse transcription to cDNA was used by the M‐MLV reverse transcriptase kit (Promega Corporation) according to the manufacturer's protocol. The primers for OCT4, Nanog, SOX2 and GAPDH were as follows: OCT4 (NM_001173531.3) forward, 5′‐CTTGAATCCCGAATGGAAAGGG‐3′ and reverse, 5′‐GTGTATATCCCAGGGTGATCCTC‐3′; GAPDH (NM_001256799.3) forward, 5′‐GGAGCGAGATCCCTCCAAAAT‐3′ and reverse, 5′‐GGCTGTTGTCATACTTCTCATGG‐3′; Nanog (NM_024865.4) forward, 5′‐TTTGTGGGCCTGAAGAAAACT‐3′ and reverse, 5′‐AGGGCTGTCCTGAATAAGCAG‐3′; and SOX2 (NM_003106.4) forward, 5′‐GCCGAGTGGAAACTTTTGTCG‐3′ and reverse, 5′‐GGCAGCGTGTACTTATCCTTCT‐3′. qPCR was performed with the SYBR‐Green Real‐Time PCR assay kit. The PCR amplification reactions were performed as follows: 1 cycle at 95°C for 5 min, followed by 35 cycles at 95°C for 12 s, 60°C for 30 s.

### 2.7 Western blot

For western blot assay, BXPC‐3 cells were plated in 6 cm dishes(1 × 10^6^ cells/dish) and treated with different concentrations of umbelliprenin or 3‐MA as indicated. After incubation for 24 h, cells were harvested and extracted by RIPA buffer lysis buffer on ice (Beyotime Institute of Biotechnology). Cell lysate was centrifuged at 14,000 rpm for 10 min and the supernatants were collected. The protein concentrations were quantified by the Quick Start Bradford Dye Reagent (Bio‐Rad). Total protein was mixed by adding 6× loading buffer and boiled for 5 min. A total of 30 μg proteins were measured by 4%–12% SDS‐PAGE (Amersham Bioscience). Following incubated with the primary and secondary antibodies, the membranes were detected by ECL (Thermo Scientific) and analyzed on a ChemiDoc XRS+(Bio‐Rad Laboratories, Inc.).

### 2.8 Transfections and Immunofluorescence microscopy

BxPC3 cells were transfected with TF‐LC3 (mRFP‐GFP‐tandem fluorescent LC3) plasmid (#21074, addgene) by using lipofectamine 3000 (Invitrogen) and seeded on coverslips at 10000/well before receiving umbelliprenin or DMSO treatment. After added umbelliprenin or DMSO for 24 h, the cells were washed with PBS and fixed with 4% paraformaldehyde and stained with DAPI (ab228549, abcam). The green and red puncta were imaged by Zeiss LSM 700 confocal microscope equipped with a 63× oil immersion objective (Zeiss GmbH).

### 2.9 Immunoprecipitation (IP) assays

Following culture in the 6 cm plates for 24 h, BxPC3 cells were transiently transfected with 4 μg of pCDNA4‐Vps34‐Flag (#24398, addgene) plasmid with Lipofectamine 3000 (Invitrogen) and then treated with umbelliprenin or DMSO. The cell lysates containing 500 μg of total protein were mixed with 1 μg of Vps34 antibody and protein A/G beads overnight. The samples were separated by SDS‐PAGE and visualized with enhanced ECL reagents.

### 2.10 Crispr‐cas9 system

Atg7‐KO stable cell line was built by using lentiCRISPRv2 (#52961, Addgene) plasmid. Targeting gRNA sequences: Atg7 gRNA 5′‐AATAATGGCGGCAGCTACGG‐3′ was cloned into the vector by *BbsI* according to molecular cloning protocol.^24^ Plasmids were transfected into BxPC3 cells by using lipofectamine 3000 (Invitrogen) and the stable colonies were selected by 1 μg/mL puromycin.

### 2.11 Xenograft model

The animal experiments and protocol were approved by the animal ethics committee of Shanghai Jiao Tong University (2021–08). A total of 20 nude mice (BALB/c; age, 4–6 weeks; weight, 18–20 g; male) were purchased from Shanghai Laboratory Animal Center and were fed in a specific pathogen‐free (SPF) room. To determine the in vivo anticancer efficacy of umbelliprenin, BxPC3 cells (4 × 10^6^ cells) were injected subcutaneously into the flank of each nude mouse. When the tumor volume reached about 100 mm^3^, the nude mice were randomly divided into five groups for different treatments (each has four mice): (i) control group (DMSO dissolved in PBS, once daily i.p.); (ii) 3‐MA group (25 mg/kg/day, i.p.); (iii) umbelliprenin group (50 mg/kg/day, i.p.); (iv) gemcitabine group (100 mg/kg, twice weekly, i.p.) and (v) umbelliprenin(50 mg/kg) +3‐MA (25 mg/kg) group (once daily, i.p.).^25^ The body weight and tumor volume were measured every 4 days. 28 days after transplanting, the mice were sacrificed and the tumor samples were isolated and measured.

### 2.12 Statistical analysis

All data shown were presented as the means ± S.D. of three independently repeated experiments. For multiple comparisons, the statistical differences were performed by one‐way ANOVA or two‐tailed *t*‐test. It was regarded to be statistically significant when *p*‐value <0.05. Graphpad Prism 6.0 was used for statistical analysis.

## 3 RESULTS

### 3.1 Umbelliprenin inhibits pancreatic cancer cell proliferation

We first explored the anti‐pancreatic cancer effect of umbelliprenin by MTT assay. After treatment with different concentrations of umbelliprenin, we found that umbelliprenin dose‐ and time‐dependently inhibited pancreatic cancer cell proliferation (Figure 1B–D). Umbelliprenin had marginally more cell growth inhibition effects on BxPC3 (IC_50_ = 45.15 ± 2.57 μg/mL) than PANC‐1 cells (IC_50_ = 47.13 ± 5.13 μg/mL) and Capan‐1 cells (IC_50_ = 51.34 ± 5.66 μg/mL) at 48 h. Therefore, we selected BxPC3 to further investigate its underlying mechanisms in pancreatic cancer cells.

### 3.2 Umbelliprenin induces apoptosis

**FIGURE 2:**
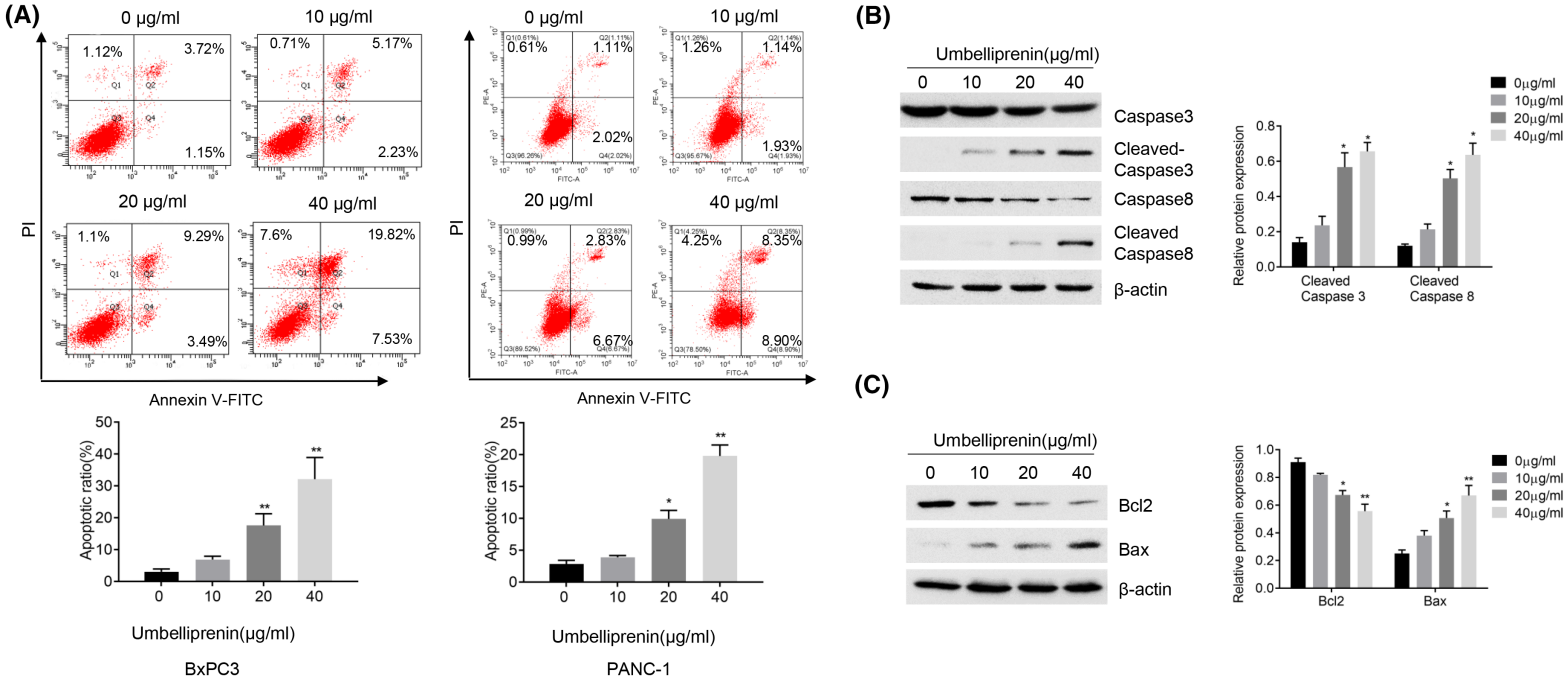
(A) Umbelliprenin‐induced apoptosis. BxPC3 and PANC‐1 cells were treated with umbelliprenin for 24 h and stained with Annexin V/PI and measured by FACS. All data shown represent the mean ± SD. *p < 0.05, **p < 0.01. (B and C) Apoptosis‐related proteins were determined from BxPC3 cells treated with umbelliprenin (0–40 μg/mL) and measured by immunoblotting as indicated. *p < 0.05, **p < 0.01.

One of the mainstays for antitumor therapy is apoptosis, thus we explored whether umbelliprenin could induce apoptosis. The FACS results showed that the percentage of apoptotic cells were increased from 4.87% (control group) to 27.35% (umbelliprenin 40 μg/mL) after umbelliprenin treatment in BxPC3 cells and from 3.13% (control group) to 17.25% (umbelliprenin 40 μg/mL) in PANC‐1 cells, respectively (Figure 2A). In addition, umbelliprenin could dose‐dependently increase the expression level of cleaved caspase‐3, caspase‐8, and the proapoptotic protein Bax while reducing the expression of antiapoptotic protein Bcl‐2, indicating that umbelliprenin could induce apoptosis in BxPC3 cells (Figure 2B,C).

### 3.3 Umbelliprenin induces autophagy

**FIGURE 3:**
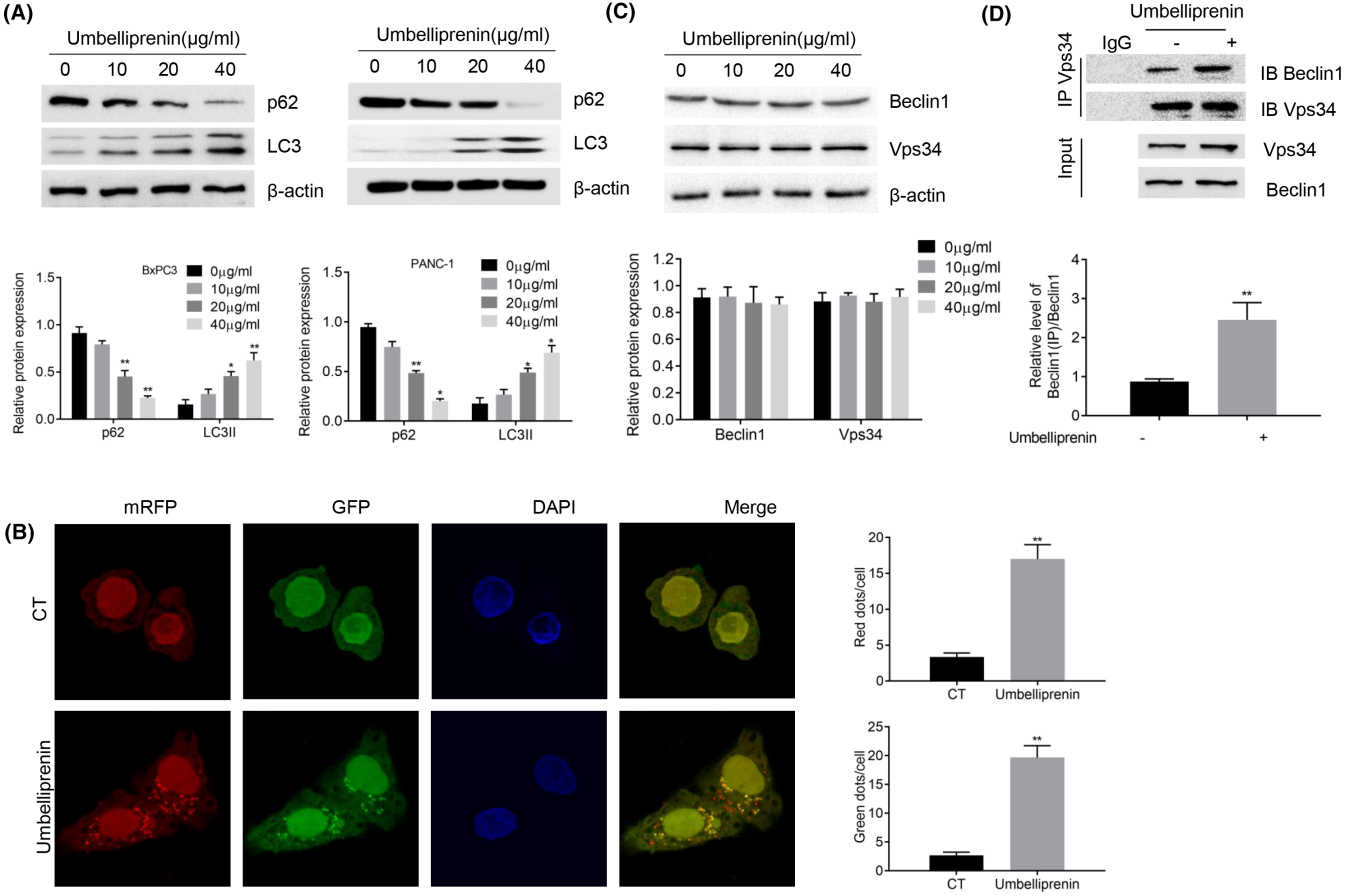
Umbelliprenin induced autophagy. (A and C) Autophagy‐related proteins were determined by immunoblotting after different concentrations of umbelliprenin (0–40 μg/mL) added into BxPC3 and PANC‐1 cells for 24 h. (B) BxPC3 cells transfected with TF‐LC3 were incubated with/without umbelliprenin for 24 h (CT = control group). Quantification of autophagosomes (yellow dots) and autolysosomes (red‐only dots). Data are represent as mean ± SEM (N = 50). Scale bars: 10 μm. (D) Cells treated with/without umbelliprenin were lysed and the endogenous Beclin1 were immunoprecipitated by using anti‐Vps34 antibody or IgG as control. **p < 0.01, ***p < 0.001.

We further examined whether umbelliprenin could trigger autophagy in BxPC3 and PANC‐1 cells by measuring the autophagic markers LC3II and p62. Immunoblotting showed that umbelliprenin dose‐dependently increased LC3II expression and p62 degradation (Figure 3A). Tf‐LC3 (mRFP‐GFP‐tandem fluorescent LC3) was used to measure the autophagic flux in BxPC3 cells, as the green or yellow dots represented early‐stage autophagosomes and the red‐only dots represented the late‐stage matured autolysosomes (GFP fluorescence was quenched in the acidic environment). The immunostaining results showed that both the yellow and red‐only dots were significantly accumulated after umbelliprenin treatment as compared with the control group (CT) (Figure 3B). During the initiation of autophagy, Beclin1 can interact with Vps34 to form PI3K complex. This complex then promotes Vps34 lipid kinase activity and generates phosphatidylinositol‐3‐phosphate (PI3P). We therefore measured whether umbelliprenin regulated the expression level of Vps34 and Beclin1. The results showed that umbelliprenin could not affect Vps34 and Beclin1 expression (Figure 3C); however, umbelliprenin significantly enhanced the interaction between Vps34 and Beclin1 (Figure 3D).

### 3.4 Umbelliprenin‐induced autophagy attenuates apoptosis

**FIGURE 4:**
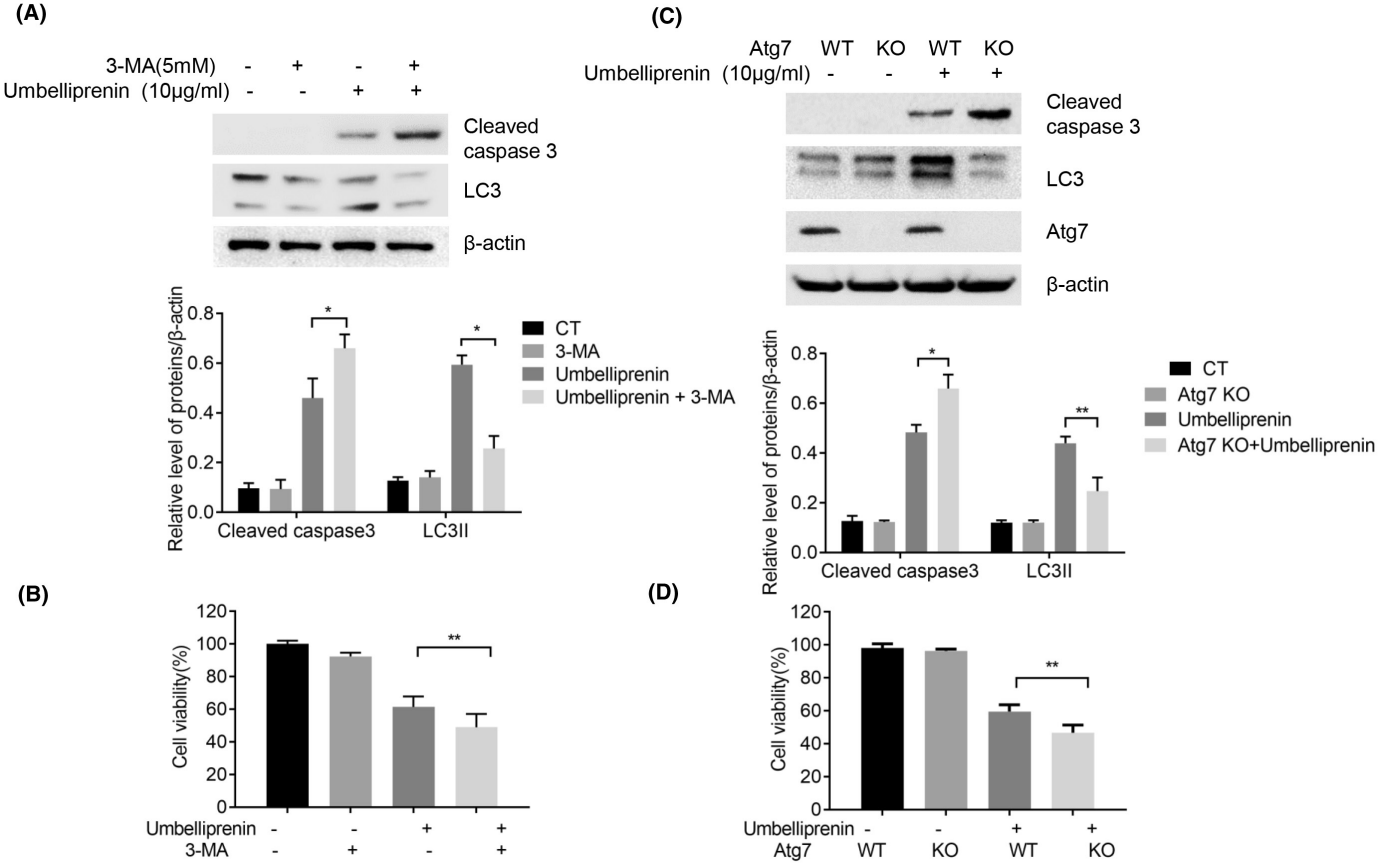
Umbelliprenin‐induced autophagy attenuated apoptosis. BxPC3 cells incubated with umbelliprenin (10 μg/mL) and/or 3‐MA (5 mM) were used for western blot assay (A) and MTT assay (B) as indicated. Atg7‐WT and Atg7‐KO BxPC3 cells were treated with umbelliprenin then western blot assay (C) and MTT assay (D) were performed as indicated. *p < 0.05, **p < 0.01.

The relationship between autophagy and apoptosis seems context dependent as autophagy can either prevent or promote apoptosis. To explore the role of umbelliprenin‐induced autophagy in BxPC3 cells, we co‐incubated umbelliprenin with 3‐MA, an autophagy inhibitor. As shown in Figure 4A, the accumulation of LC3II was decreased after 3‐MA added in; however, umbelliprenin plus 3‐MA could significantly increase the cleavage of caspase 3 as compared with umbelliprenin or 3‐MA treatment alone. Consistently, the cell growth was greatly inhibited after combination treatment (Figure 4B). To further verify the role of umbelliprenin‐induced autophagy in BxPC3 cells, we generated Atg7‐negative BxPC3 cells (KO) or BxPC3 cells with empty CRISPR/Cas9 plasmid as control (WT). We found that apoptosis and cell growth inhibition induced by umbelliprenin were an augment in Atg7 KO cells but not in Atg7 WT cells, suggesting that inhibition of autophagy increased umbelliprenin‐induced apoptosis in BxPC3 cells (Figure 4C,D).

### 3.5 Umbelliprenin inhibits pancreatic cancer cell stemness

**FIGURE 5:**
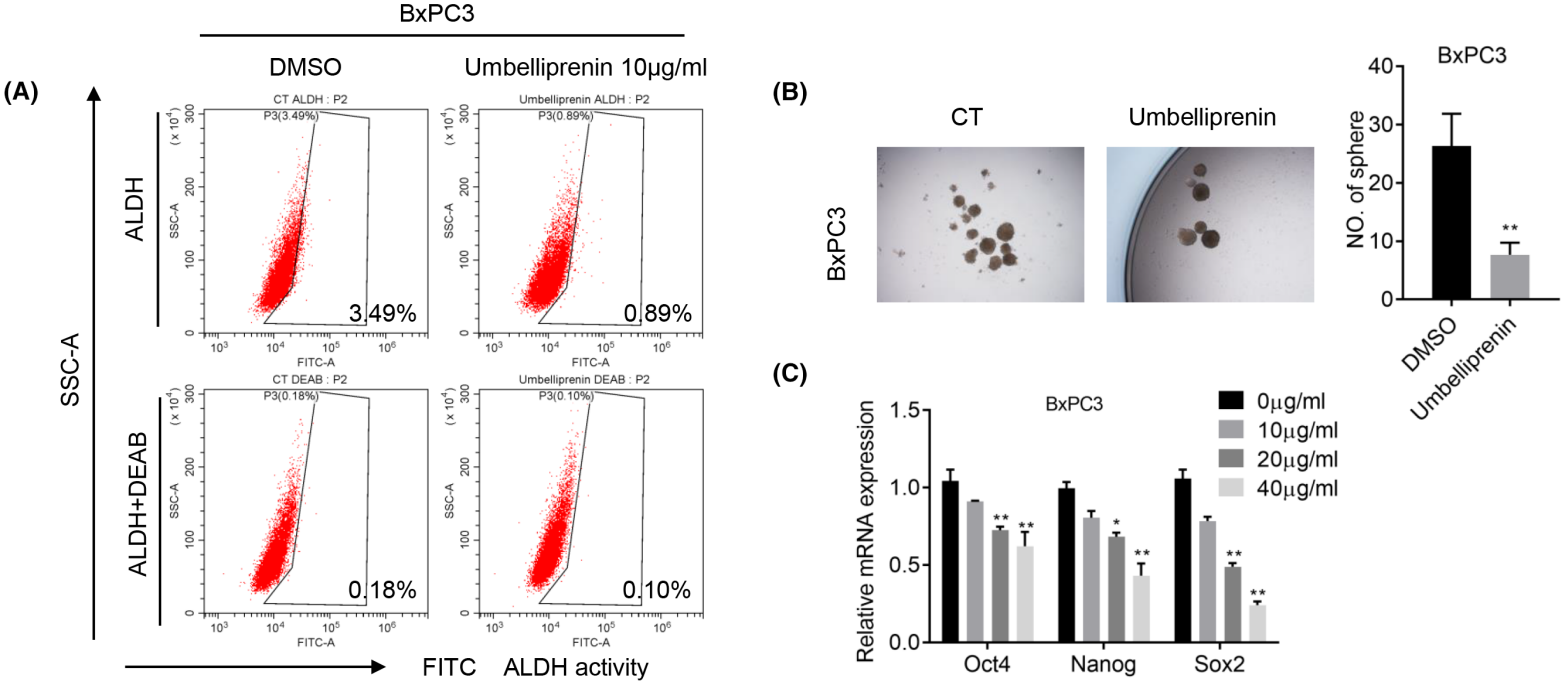
Umbelliprenin reduced pancreatic cancer cell stemness. BxPC3 cells were treated with/without umbelliprenin (10 μg/mL). (A) ALDEFLUOR assay was carried out. (B) The cells self‐renewal ability was evaluated by tumor‐sphere formation assay. (C) The mRNA level of stem cell markers was analyzed by reverse transcription‐quantitative PCR. *p < 0.05, **p < 0.01, and ***p < 0.001.

To determine whether umbelliprenin regulated pancreatic cancer cell stemness, BxPC3 cells were treated with umbelliprenin and followed by ALDEFLOUR assay. It showed that the proportion of ALDH+ cells in the umbelliprenin group were greatly decreased when treated with umbelliprenin (Figure 5A). In addition, the mammosphere formation assays showed that treatment with umbelliprenin significantly reduced the size and the number of formed mammospheres in BxPC3 cells (Figure 5B). Moreover, NANOG, OST4, and SOX2 mRNA expression levels were all downregulated after umbelliprenin added in (Figure 5C). Therefore, it revealed that umbelliprenin significantly suppressed cancer cell stemness in pancreatic cancer.

### 3.6 Umbelliprenin inhibits Akt signal pathway in pancreatic cancer

**FIGURE 6:**
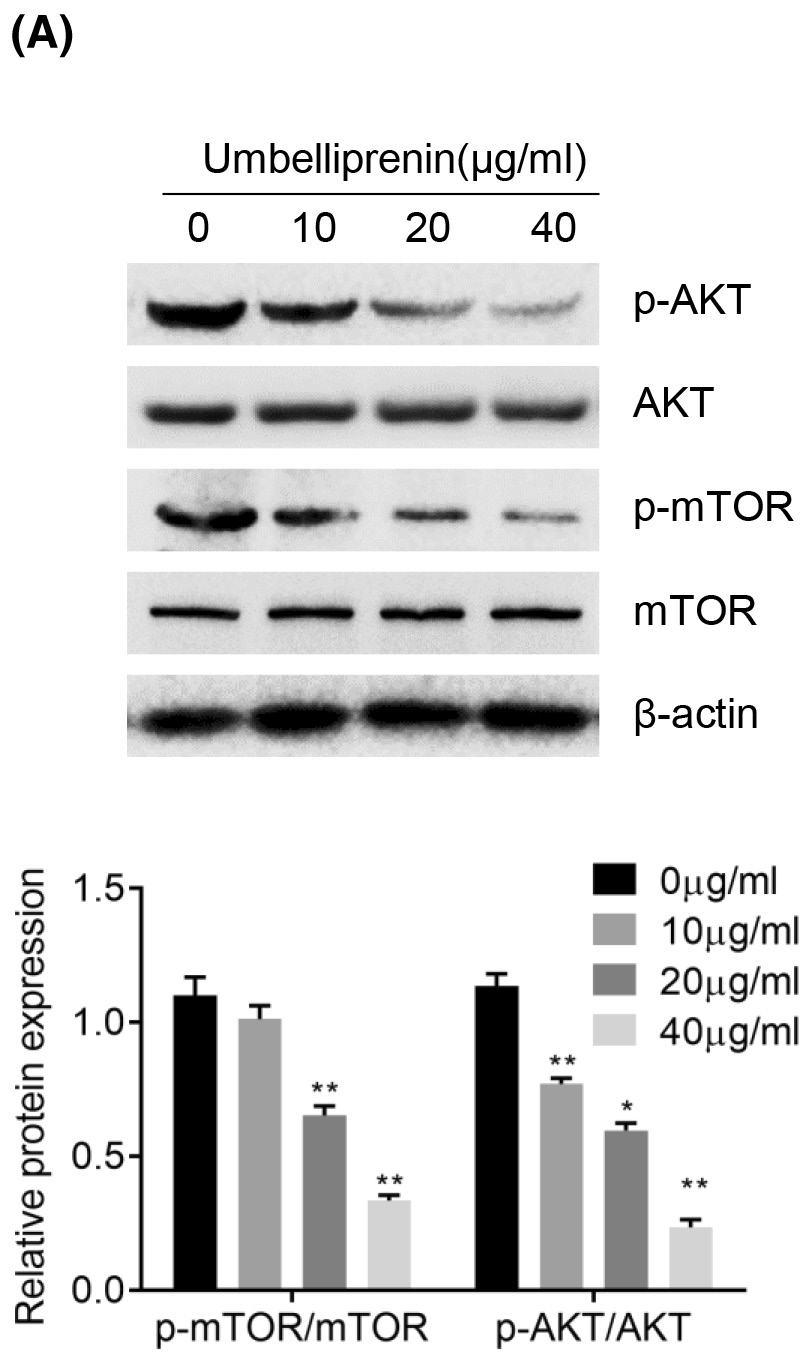
Umbelliprenin inhibited Akt signal pathway. (A) BxPC3 cells were treated with different concentrations of umbelliprenin (0–40 μg/mL). Then the expression of Akt signal pathway were examined. *p < 0.05, **p < 0.01.

PI3K/Akt signal pathway is one of the major signal pathways in the regulation of both apoptosis and autophagy. Our results showed that umbelliprenin decreased Akt and mTOR phosphorylation level, while the total protein levels of Akt and mTOR were not significantly changed (Figure 6A). These results indicated that Akt/mTOR signaling pathway was involved in umbelliprenin‐induced autophagy.

### 3.7 Umbelliprenin regulates Notch1 signal pathway in pancreatic cancer

**FIGURE 7:**
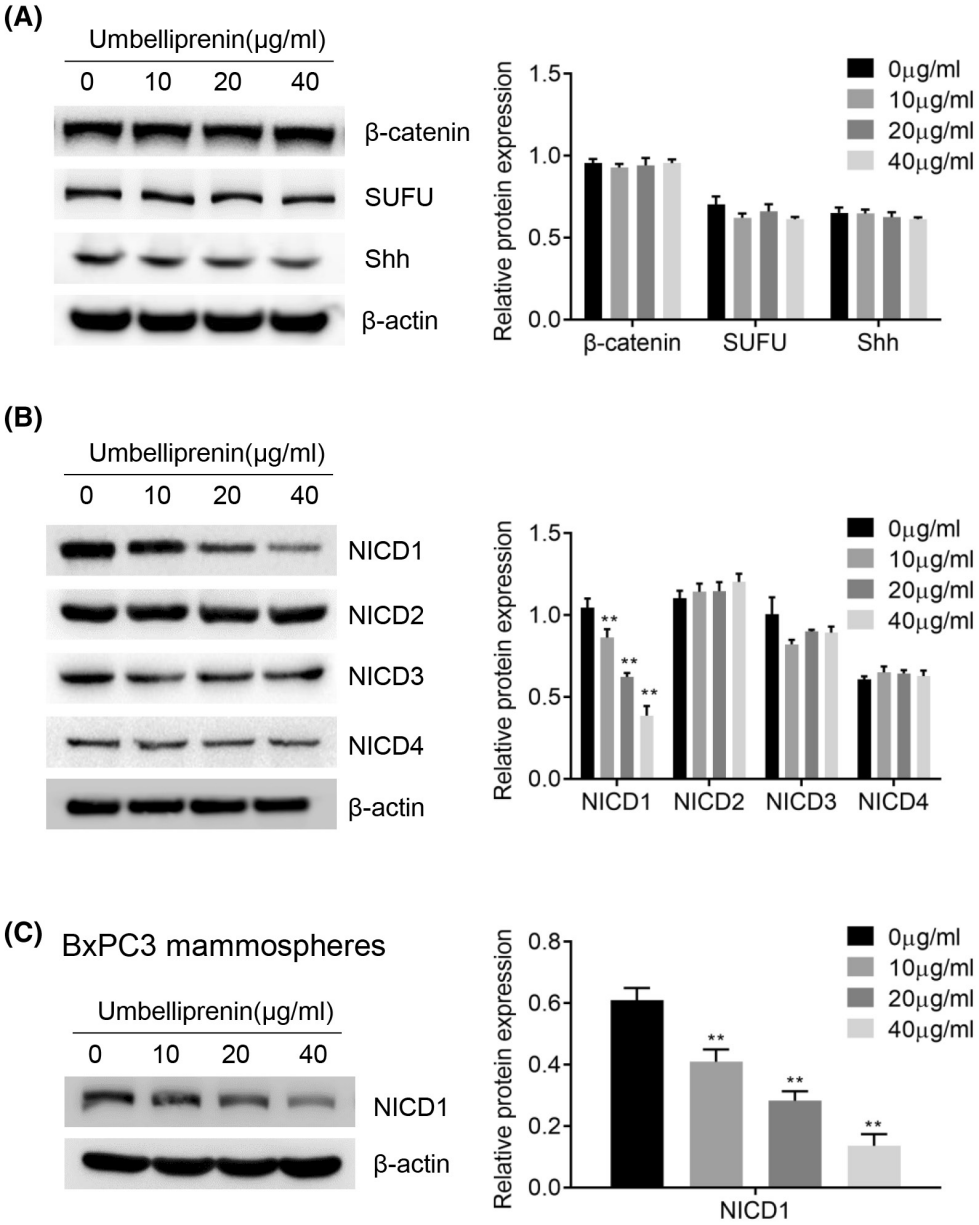
Umbelliprenin downregulates Notch1 signal pathway in pancreatic cancer. BxPC3 cells were treated with different concentrations of umbelliprenin (0–40 μg/mL). Protein level of β‐catenin, Hedgehog, and Notch pathways were measured by western blot assay in BxPC3 cels (A and B) or mammosphere (C). **p < 0.01.

NOTCH, β‐catenin, and Hedgehog signaling pathways were important regulators to maintain CSCs. We found that umbelliprenin treatment did not change the protein expression level of β‐catenin, Shh, and SUFU (Figure 7A), indicating that Wnt/β‐catenin and Hedgehog signaling pathways are not involved in the umbelliprenin‐mediated inhibition of pancreatic cancer cell stemness. However, umbelliprenin could significantly reduce the expression of Notch1 intracellular domain (NICD1) but not NICD2, NICD3, and NICD4 in BxPC3 cells (Figure 7B). In addition, the NICD1 expression in BxPC3 mammospheres was decreased after umbelliprenin treatment (Figure 7C). These results indicated the importance of Notch1 signaling pathway in the umbelliprenin‐regulated inhibition of pancreatic cancer cell stemness.

### 3.8 Umbelliprenin inhibits tumor growth in vivo

**FIGURE 8:**
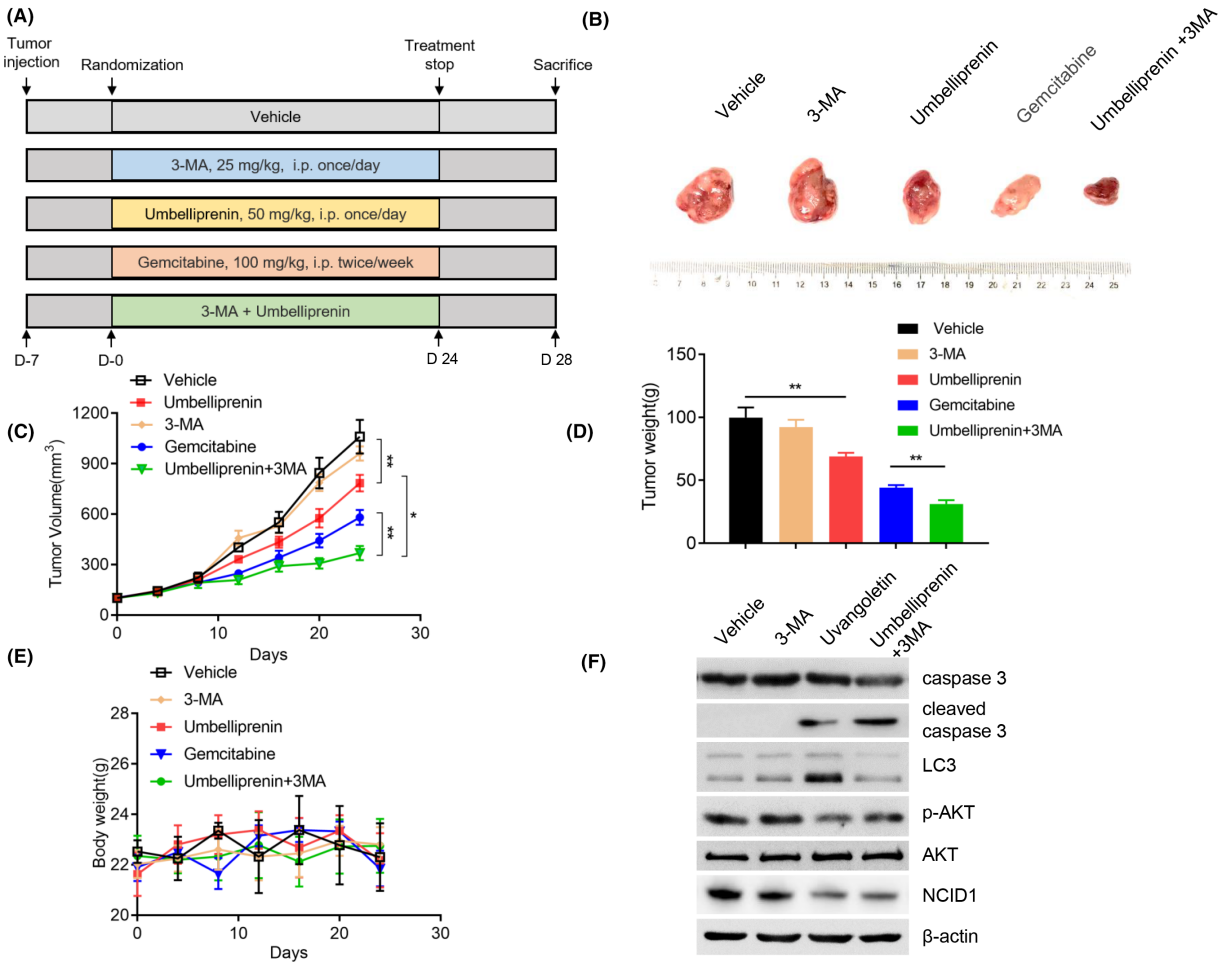
Umbelliprenin inhibited tumor growth in vivo. BxPC3 cells were injected subcutaneously into the flank of each nude mouse. When the tumor volume reached about 100 mm^3^, the nude mice were assigned into five groups. (A) flow chart representation of experimental design for in vivo studies. (B) Representative tumors removed from each treatment groups were photographed (C and D) Tumor volume and weight of each group were measured. (E) Body weight of each group were measured. (F) Typical proteins were detected by immunoblotting in tumor samples. *p < 0.05, **p < 0.01.

To further explore whether umbelliprenin could inhibit pancreatic tumor growth in vivo, a xenograft model was established using BxPC3 cells. The treatment schema is summarized in Figure 8A. BxPC3 cells were injected subcutaneously into the flank of each nude mouse. When the tumor volume reached about 100 mm^3^, the nude mice were assigned into five groups. We found that umbelliprenin suppressed BxPC3 tumor growth as compared with control group. Umbelliprenin in combination with 3‐MA greatly reduced the tumor burden compared with the umbelliprenin group or even the gemcitabine group (Figure 8B–D). However, the body weight of these mice had no significant difference (Figure 8E). Moreover, we measured the expression levels of apoptosis, autophagy‐related proteins, and Notch1 pathway in these tumor samples. It revealed that umbelliprenin upregulated the expression of LC3II, the cleavage of caspase 3, downregulated the expression level of p‐Akt and NICD1 (Figure 8F). Collectively, these data suggested that umbelliprenin induced cell apoptosis and autophagy inhibits cancer cell stemness in vivo. The combination of 3‐MA with umbelliprenin significantly reduced tumor burden as compared with gemcitabine or umbelliprenin treatment alone.

## 4 DISCUSSION

Coumarins and their derivatives are ubiquitous in nature and exert various anticancer mechanisms. Coumarin derivatives like irosustat are under clinical trials to treat cancer, suggesting their potential in the development of novel drugs.^26^ Umbelliprenin is a sesquiterpene coumarin extracted from *A. absinthium* It has also been found in various common dietary sources such as celery, Coriandrum sativum, and citrus limon.^27^ Previous studies demonstrated that umbelliprenin inhibits cancer cell proliferation in various cancer cells. However, its antitumor effect in pancreatic cancer has not been reported. In the current study, we found that umbelliprenin dose‐ and time‐dependently inhibited pancreatic cancer cell and tumor growth in vitro and in vivo.

Apoptosis is recognized as prototypical Type I programmed cell death, which is regulated by both the intrinsic and extrinsic pathways. Caspases are cleaved and activated during the apoptotic process. The proapoptotic protein Bax and antiapoptotic protein Bcl‐2 can regulate the apoptotic mitochondrial pathways by regulation the release of cytochrome c from mitochondria.^28^ In this study, we found that umbelliprenin increased the cleavage of caspase3 and caspae8 and Bax; while decreasing the expression of Bcl‐2. Furthermore, the percentage of Annexin V/PI‐positive cells was upregulated after umbelliprenin treatment, suggesting that umbelliprenin‐induced apoptosis in BxPC3 cells through both intrinsic and extrinsic pathways. Consistently, previous studies showed that umbelliprenin could induce apoptosis in various cancer cells.^19^, ^20^, ^21^, ^29^

Autophagy is an evolutionarily conserved catabolic process induced by various cellular stress, which inhibits cell damage and helps tumor cell survival. During this process, LC3 was conjugated with phosphatidylethanolamine (PE) to form lapidated LC3 (LC3II) and stably associated with the autophagosome membrane. Therefore, LC3II is widely used as an autophagic biomarker. Another autophagic biomarker is p62, which interacts with LC3 and transports into the autolysosome for degradation.^30^ The Vps34 complex, consisting of Vps34, Beclin1, and Vps15, plays a pivotal role in the initiation of autophagosome formation. In this complex, Bcelin1 interacted with Vps34 to promote its lipid kinase activity, thereby promoting the synthesis of PI3P and increasing autophagosome formation.^31^ Our findings showed that umbelliprenin could increase LC3II accumulation, promote p62 degradation in vitro and in vivo. Although umbelliprenin could not increase Vps34 or Beclin1 expression, it could significantly increase Vps34‐Beclin1 interaction. These results suggested that umbelliprenin induced autophagy in BxPC3 cells.

The relationship between coumarin‐induced autophagy and apoptosis are complicated. Coumarins such as compound 8b (a hybrid of coumarin and phenylsulfonylfuroxan), daphnetin, and urolithins could induce autophagy to help cell survival^32^, ^33^, ^34^; while coumarins and their derivatives such as Psoralidin, 3‐benzyl coumarin seco‐B‐ring derivative and *Ferulin C* induced autophagic cell death.^35^, ^36^, ^37^ Thus, it is necessary to examine the role of umbelliprenin‐induced autophagy in pancreatic cancer. In this study, we showed that umbelliprenin‐induced cell growth inhibition and apoptosis was augmented after blocking autophagy by 3‐MA. Moreover, the number of caspase3 cleavage was much more in Atg7‐KO BxPC3 cells than in Atg7‐WT BxPC3 cells after umbelliprenin treated. These results suggested that umbelliprenin‐induced autophagy plays a cyto‐protective role in pancreatic cancer cells.

Pancreatic CSCs have been involved in tumor initiation, metastases, recurrence, and drug resistance. Stemness‐associated biomarkers like SOX2, OCT4, Nanog, and increased ALDH activity are responsible for cancer stem cell self‐renew and differentiation and correlated with poor prognosis in pancreatic cancer patients.^38^ In the present study, we observed that umbelliprenin greatly decreased the subpopulation of ALDH+ pancreatic CSCs and reduced the number and size of formed stem cell spheres. Umbelliprenin treatment significantly downregulated the mRNA of Oct4, Nanog, and SOX2, suggesting umbelliprenin as a putative pancreatic cancer stem cell killing drug.

Akt signal pathway plays a critical role in cell cycle, apoptosis and autophagy.^39^ Many antitumor chemicals are reported to induce autophagy and apoptosis in cancer cells through Akt signal pathway.^40^, ^41^ In the present study, we showed that umbelliprenin significantly reduced the phosphorylation levels of AKT and mTOR. These results demonstrated that umbelliprenin‐mediated induction of apoptosis and autophagy might be involved in Akt signaling pathway inhibition.

We also revealed that umbelliprenin exhibited the potential in decreasing pancreatic CSCs by inhibiting Notch1 signal pathway. Notch signal pathway plays a critical role in the mediation of the pancreatic CSC phenotype during cancer progression.^42^ While silencing Notch1 results in the inhibition of cell proliferation, reduction of ALDH+ cell, and induction of apoptosis and gemcitabine resistance in pancreatic cancer.^43^ We showed that umbelliprenin treatment significantly reduced NICD1 expression in BxPC3 cells but not NICD2, NICD3, or NICD4 expression. In addition, NICD1 protein expression was downregulated in BxPC3 mammospheres after umbelliprenin added in. These results suggested that Notch1 signaling pathway played a critical role in umbelliprenin‐mediated pancreatic cancer cell stemness.

## 5 CONCLUSION

In summary, our study showed that umbelliprenin significantly inhibited pancreatic tumor cell growth in vitro and in vivo. In addition, umbelliprenin could induce apoptosis and cyto‐protective autophagy by blocking Akt signaling pathway. Umbelliprenin could diminish pancreatic CSCs by inhibiting Notch1 signaling pathway. It suggests a promising application of umbelliprenin combinate with 3‐MA as an adjunctive agent for the treatment of pancreatic cancer.

## AUTHOR CONTRIBUTIONS

**Hongcheng Wang:** Conceptualization (equal); funding acquisition (lead); investigation (equal); methodology (equal); writing – original draft (lead). **Yongzhi Liu:** Conceptualization (equal); data curation (equal); formal analysis (equal); investigation (equal); methodology (equal). **Yiwei Wang:** Data curation (supporting); formal analysis (supporting); methodology (supporting). **Xu Ting:** Formal analysis (supporting); methodology (supporting). **Guanggai Xia:** Data curation (supporting); investigation (supporting); methodology (supporting). **Xinyu Huang:** Conceptualization (supporting); investigation (supporting); supervision (lead); writing – review and editing (lead).

## CONFLICT OF INTEREST STATEMENT

The authors declare no conflicts of interest.

## Data Availability

Data available on request from the authors.
